# Tenecteplase Thrombolysis in Posterior Circulation Stroke

**DOI:** 10.3389/fneur.2021.678887

**Published:** 2021-08-06

**Authors:** Fana Alemseged, Bruce C. V. Campbell

**Affiliations:** Department of Medicine and Neurology, Royal Melbourne Hospital, University of Melbourne, Parkville, VIC, Australia

**Keywords:** tenecteplase, basilar artery occlusion, alteplase, posterior circulation stroke, thrombolytic agent

## Abstract

One in five ischaemic strokes affects the posterior circulation. Basilar artery occlusion is a type of posterior circulation stroke associated with a high risk of disability and mortality. Despite its proven efficacy in ischaemic stroke more generally, alteplase only achieves rapid reperfusion in ~4% of basilar artery occlusion patients. Tenecteplase is a genetically modified variant of alteplase with greater fibrin specificity and longer half-life than alteplase, which can be administered by intravenous bolus. The single-bolus administration of tenecteplase vs. an hour-long alteplase infusion is a major practical advantage, particularly in “drip and ship” patients with basilar artery occlusion who are being transported between hospitals. Other practical advantages include its reduced cost compared to alteplase. The EXTEND-IA TNK trial demonstrated that tenecteplase led to higher reperfusion rates prior to endovascular therapy (22 vs. 10%, non-inferiority *p* = 0.002, superiority *p* = 0.03) and improved functional outcomes (ordinal analysis of the modified Rankin Scale, common odds ratio 1.7, 95% CI 1.0–2.8, *p* = 0.04) compared with alteplase in large-vessel occlusion ischaemic strokes. We recently demonstrated in observational data that tenecteplase was associated with increased reperfusion rates compared to alteplase prior to endovascular therapy in basilar artery occlusion [26% (*n* = 5/19) of patients thrombolysed with TNK vs. 7% (*n* = 6/91) thrombolysed with alteplase (RR 4.0 95% CI 1.3–12; *p* = 0.02)]. Although randomized controlled trials are needed to confirm these results, tenecteplase can be considered as an alternative to alteplase in patients with basilar artery occlusion, particularly in “drip and ship” patients.

## Introduction

One in five ischaemic strokes affects the posterior circulation (1). This type of stroke is associated with a high risk of recurrence, disability, and mortality (2). It has been over 25 years since the publication of the National Institute of Neurological Disorders and Stroke (NINDS) tPA trial (3), the first large positive clinical trial of recombinant tissue plasminogen activator (tPA or alteplase) in ischaemic stroke patients. Despite accounting for 20% of all strokes (1), only 5% of patients from the NINDS study (3) had a posterior circulation stroke and these patients were underrepresented in most of the positive clinical trials of alteplase (4–7). However, several observational studies have demonstrated comparable efficacy and safety profiles in patients with anterior and posterior circulation stroke treated with alteplase. Several studies have also suggested a lower risk of haemorrhagic complications in posterior circulation compared to anterior circulation strokes (8–13). The lower risk of haemorrhagic transformation in posterior circulation stroke may be explained by a stronger tolerance to the ischaemic insult in the posterior circulation territory, likely due to its greater proportion of white matter and collateral pathways, particularly in the brainstem (14). Furthermore, the lower infarct volume in posterior circulation stroke compared to anterior circulation stroke may result in lower bleeding risk in these patients (15). Basilar artery occlusion is a type of posterior circulation stroke associated with a high risk of disability and mortality (16, 17). However, clinical outcomes in basilar artery occlusion improve if reperfusion is achieved. Despite its proven efficacy in ischaemic stroke more generally, alteplase only achieves rapid reperfusion in ~4% of basilar artery occlusion patients (18).

## Tenecteplase In Ischaemic Stroke

Tenecteplase is a genetically modified variant of alteplase with greater fibrin specificity and longer half-life than alteplase (22 min for tenecteplase compared to 3–5 min for alteplase) (19), which can be administered by intravenous bolus without the need for the 1-h infusion of alteplase. Tenecteplase has been approved for acute myocardial infarction and was demonstrated to be superior to alteplase (20, 21). The first trial testing tenecteplase in stroke was an open-label, dose-escalation safety study comparing 0.1, 0.2, 0.4, and 0.5 mg/kg in *n* = 88 ischaemic stroke patients within 3 h (22). Although enrolment into the dose used for myocardial infarction (0.5 mg/kg) was closed prematurely due to the high risk of symptomatic intracranial hemorrhage, the doses of 0.1 to 0.4 mg/kg appeared safe in ischaemic stroke (22). Nonetheless, the 0.4-mg/kg dose tier in a subsequent phase 2b study was terminated due to safety concerns (23). In 2012, Parsons et al. completed a randomized phase IIb study in which they compared tenecteplase 0.1 mg/kg (*n* = 25 patients) and 0.25 mg/kg (*n* = 25 patients) to alteplase 0.9 mg/kg (n = 25 patients) in a cohort of ischaemic stroke patients with large-vessel occlusion and visually assessed salvageable tissue on CT perfusion, within 6 h of symptom onset (24). In this trial, which preceded the use of endovascular thrombectomy, the pooled tenecteplase groups had greater reperfusion (*p* = 0.004) and better outcomes (modified Rankin Score 0–2, 72 vs. 44%, p = 0.02) than the alteplase group. Tenecteplase was associated with increased reperfusion, early neurological improvement, and improved 3-month functional outcome with a strong dose-dependent relationship, with the 0.25-mg/kg dose achieving better efficacy outcomes compared to 0.1 mg/kg, and no increase in symptomatic intracerebral hemorrhage (24). A subsequent phase II trial compared tenecteplase 0.25 mg/kg to alteplase 0.9 mg/kg in n = 104 ischaemic stroke patients within 4.5 h of symptom onset. No significant differences were found for the primary endpoint of percentage of penumbra salvaged (68% [SD 28] in the tenecteplase group vs. 68% [SD 23] in the alteplase group; mean difference 1.3% [95% CI −9.6 to 12.1]; *p* = 0·81) (25). However, a subsequent pooled analysis of these two trials demonstrated that treatment with tenecteplase was associated with greater early clinical improvement (median National Institutes of Health Stroke Scale score change: tenecteplase, 6; alteplase, 1; *p* < 0.001) and better functional outcomes (modified Rankin scale score 0–1: odds ratio, 2.33; 95% CI, 1.13–5.94; *p* = 0.032) than those treated with alteplase, with the greatest benefit seen in patients with a CT perfusion-defined target mismatch (26). Furthermore, patients with anterior circulation large-vessel occlusion treated with tenecteplase had higher recanalization rates at 24 h (71% for tenecteplase vs. 43% for alteplase, *p* = 0.001) and significantly better functional outcomes (modified Rankin scale score 0–1: odds ratio 4.82, 95% confidence interval 1.02–7.84, *p* = 0.05) than patients treated with alteplase (27). Patients with basilar artery occlusion were not included in these trials.

The Tenecteplase vs. Alteplase before Endovascular Therapy for Ischemic Stroke (EXTEND-IA TNK) trial compared tenecteplase 0.25 mg/kg to alteplase prior to endovascular therapy in n = 202 patients with large-vessel occlusion (28). In this trial, tenecteplase led to higher reperfusion rates prior to endovascular therapy (22 vs. 10%, non-inferiority *p* = 0.002, superiority *p* = 0.03) and improved functional outcomes (ordinal analysis of the modified Rankin Scale, common odds ratio 1.7, 95% CI 1.0–2.8, *p* = 0.04) compared with alteplase in large-vessel occlusion ischaemic strokes. Subsequently, the EXTEND-IA TNK part 2 trial compared tenecteplase 0.25 mg/kg with 0.4 mg/kg and did not find any further benefit with the higher dose for vessel recanalization or improved outcomes (29). These results clarified that the optimal dose of tenecteplase for large-vessel occlusion stroke is 0.25 mg/kg. This came after the large phase III Norwegian Tenecteplase Stroke Trial (NOR-TEST) trial, comparing tenecteplase 0.4 mg/kg to alteplase in *n* = 1,107 stroke patients recruited within 4.5 h of onset or of awakening from sleep with symptoms (30). In this trial, tenecteplase did not show superiority in improving excellent outcome (modified Rankin Scale 0–1, 64 vs. 63%, odds ratio 1.08 [95% CI 0.84–1.38, *p* = 0.52]). The dose of tenecteplase 0.4 mg/kg was considered safe as the rate of symptomatic haemorrhagic transformation was not increased (*p* = 0.70). However, this cohort of patients had a very low median baseline severity (National Institutes of Health Stroke Scale score of 4) with a high number of stroke mimics (17%), which significantly reduced the power to detect a meaningful difference between the two thrombolytic agents for both efficacy and safety (30). A subsequent meta-analysis of non-inferiority including five trials of tenecteplase vs. alteplase (31) found that tenecteplase was non-inferior to alteplase for all clinical efficacy measures (modified Rankin Scale 0–1, 0–2, and ordinal analysis) as well as symptomatic haemorrhagic transformation, regardless of the dose being 0.25 mg/kg or 0.4 mg/kg or the need for endovascular therapy for large-vessel occlusion (31). No randomized controlled trial has ever investigated the effect of tenecteplase in a cohort of patients with posterior circulation stroke (with or without large-vessel occlusion). Zhong et al. recently demonstrated that the routine use of tenecteplase for stroke thrombolysis in New Zealand was feasible and had comparable safety profile and outcome to alteplase. This real-world observational study has found that tenecteplase was also safely implemented in two small regional stroke centers less experienced in stroke reperfusion treatment (32). However, the number of posterior circulation stroke patients treated with tenecteplase was not reported in this study. The 2019 American Heart Association/American Stroke Association guidelines endorsed class IIB recommendations for tenecteplase for patients with large-vessel occlusion (33). The Australian Stroke guidelines support tenecteplase as a reasonable alternative to alteplase in patients with large-vessel occlusion (strong recommendation) and non-large-vessel occlusion (weak recommendation) ischaemic stroke who meet specific clinical and brain imaging eligibility criteria (34). Ongoing phase 3 trials comparing 0.25 mg/kg tenecteplase vs. alteplase include Tenecteplase vs. Alteplase for Stroke Thrombolysis Evaluation (TASTE) in stroke patients eligible for intravenous thrombolysis with target mismatch on computed tomography perfusion imaging, the Alteplase-Tenecteplase Trial Evaluation for Stroke Thrombolysis (ATTEST-2) trial, and the Alteplase Compared to Tenecteplase in Patients With Acute Ischemic Stroke (AcT) trial enrolling patients based on non-contrast CT alone (Table 1). In Scandinavia, The Norwegian Tenecteplase Stroke Trial 2 (NOR-TEST 2) is enrolling patients 0–4.5 h on the basis of non-contrast CT using 0.40 mg/kg tenecteplase. These studies will provide Level 1 evidence on the use of tenecteplase in stroke patients within 4.5 h. Although only a small proportion of patients with posterior circulation stroke may be enrolled in these studies, the results of these trials will likely be applied to all stroke patients, regardless of infarct topography (as occurred with previous alteplase trials). Nonetheless, further studies to investigate the safety and efficacy of tenecteplase in posterior circulation stroke patients (with and without large-vessel occlusions) are warranted. Further tenecteplase randomized-controlled trials are ongoing (Table 1).

**Table 1:** Ongoing tenecteplase randomized controlled trials.

	**Number of anticipated enrolled patients**	**Posterior circulation stroke patients**	**Primary outcome**	**Primary hypothesis**	**Clinicaltrials.gov registration (or Australian New Zealand registration) number**
TASTE	*n* = 1,024	YES	mRS 0–1 (no disability) at 90 days	Tenecteplase 0.25 mg/kg is superior to alteplase 0.9 mg/kg within 4.5 h after symptom onset in patients with acute ischaemic stroke eligible for intravenous thrombolysis and with target mismatch on computed tomography perfusion imaging	ACTRN12613000243718
ATTEST-2	*n* = 1,870	YES	Ordinal mRS analysis at 90 days	Tenecteplase 0.25 mg/kg is superior to alteplase 0.9 mg/kg within 4.5 h after symptom onset in patients with acute ischaemic stroke eligible for intravenous thrombolysis	NCT02814409
AcT	*n* = 1,600	YES	mRS 0–1 (no disability) at 90 days	Tenecteplase 0.25 mg/kg is non-inferior to alteplase 0.9 mg/kg within 4.5 h after symptom onset in patients with acute ischaemic stroke eligible for intravenous thrombolysis	NCT03889249
NOR-TEST2	*n* = 1,342	YES	mRS 0–1 (no disability) at 90 days	Tenecteplase 0.4 mg/kg is superior to alteplase 0.9 mg/kg in patients with acute ischaemic stroke treated within 4.5 h after symptom onset (or after awakening with stroke symptoms)	NCT03854500
TEMPO-2	*n* = 1,274	YES	Return to baseline mRS at 90 days	Tenecteplase 0.25 mg/kg is superior to standard of care in minor ischaemic stroke patients with proven acute symptomatic intracranial occlusion within 12 h after symptom onset	NCT02398656
ETERNAL-LVO	*n* = 740	NO	mRS 0–1 (no disability) or return to baseline mRS at 90 days	Tenecteplase 0.25 mg/kg is superior to current best practice in acute ischaemic stroke patients with a large-vessel occlusion and penumbral tissue on multimodal CT or MRI within 24 h after symptom onset	NCT04454788
TIMELESS	*n* = 456	NO	Ordinal mRS analysis at 90 days	Tenecteplase 0.25 mg/kg is superior to placebo in patients with acute ischaemic stroke with a large-vessel occlusion and penumbral tissue between 4.5 and 24 h after symptom onset	NCT03785678
TWIST	*n* = 600	YES	Ordinal mRS analysis at 90 days	Tenecteplase 0.25 mg/kg is superior to best standard treatment in acute ischaemic stroke patients within 4.5 h of awakening with stroke symptoms	NCT03181360
TASTE-A	*n* = 80	YES	Volume of lesion on CT Perfusion performed on arrival at the receiving hospital	Tenecteplase 0.25mg/kg is superior to alteplase 0.9mg/kg in acute ischaemic stroke patients eligible for intravenous thrombolysis and attended by a mobile stroke unit	NCT04071613

*mRS, modified Rankin Scale score; TASTE, Tenecteplase vs. Alteplase for Stroke Thrombolysis Evaluation Trial; ATTEST-2, Alteplase-Tenecteplase Trial Evaluation for Stroke Thrombolysis trial; AcT, Alteplase Compared to Tenecteplase in Patients With Acute Ischemic Stroke trial; NOR-TEST2, The Norwegian Tenecteplase Stroke Trial 2; TEMPO-2, TNK-tPA vs. Standard of Care for Minor Ischemic Stroke With Proven Occlusion Trial; ETERNAL-LVO, Extending the Time Window for Tenecteplase by Effective Reperfusion in Patients With Large Vessel Occlusion Trial; TIMELESS, Tenecteplase in Stroke Patients Between 4.5 and 24 h Trial; TWIST, Tenecteplase in Wake-up Ischaemic Stroke Trial; TASTE-A, Tenecteplase vs. Alteplase for Stroke Thrombolysis Evaluation Trial in the Ambulance*.

## Tenecteplase In Basilar Artery Occlusion

The use of tenecteplase in basilar artery occlusion has been mostly described in case reports (35, 36). The EXTEND-IA TNK trial (28, 29) was the only tenecteplase trial including patients with basilar artery occlusion. However, it was unclear whether its findings can be extrapolated to basilar artery occlusion as only six patients were included with no difference in the primary outcome (one-third had reperfusion at initial angiography in each treatment arm).

We recently presented the first series of patients with basilar artery occlusion treated with tenecteplase (37). Our findings suggest that tenecteplase may be associated with increased reperfusion rates in comparison with alteplase in patients with basilar artery occlusion, with rates of reperfusion similar to the 22% with tenecteplase and 10% with alteplase reported in the EXTEND-IA TNK trial (28). In our study including *n* = 110 patients with basilar artery occlusion, reperfusion occurred in 26% (*n* = 5/19) of patients treated with tenecteplase vs. 7% (n = 6/91) treated with alteplase (RR 4.0, 95% CI 1.3–12; *p* = 0.02), obviating the need for endovascular therapy. This occurred despite shorter thrombolysis-to-arterial-puncture time in the tenecteplase-treated patients (48[IQR 40–71] min) vs. alteplase-treated patients (110[IQR 51–185]min, p = 0.004). No difference in symptomatic intracranial hemorrhage was observed (0/19(0%) TNK, 1/91(1%) alteplase, *p* = 0.9). In contrast to EXTEND-IA TNK (28), functional outcomes were similar in the two treatment groups but our study was underpowered to detect such differences. Nonetheless, there was a non-significant trend toward higher 3-month excellent outcomes in patients treated with tenecteplase (modified Rankin Scale 0–1 47 vs. 37%, p = 0.09) compared to alteplase, which did not translate into better outcomes after multivariable analysis adjusted for age and NIHSS (adjusted risk ratio 1.6, 95% CI 0.9–2.7; *p* = 0.1). However, patients treated with tenecteplase were older than those treated with alteplase, likely due to more recent broader age selection criteria for reperfusion therapies, and tended to have higher baseline NIHSS scores (20 (IQR 5–32) for tenecteplase-treated patients vs. 15 (IQR 7–32) for alteplase-treated patients, *p* = 0.9). These differences in baseline characteristics would favor improved functional outcomes in the alteplase group. Therefore, our findings may represent a conservative estimate of the clinical benefits associated with tenecteplase. Interestingly, in a recent meta-analysis including five tenecteplase trials (*n* = 1,585), a greater effect of tenecteplase was detected when excellent outcomes were used as primary endpoint [(modified Rankin scale 0–1, crude cumulative rates of disability-free 57.9% tenecteplase vs. 55.4% alteplase; risk difference 4% (95% CI, −1% to 8%)] compared to good outcomes (modified Rankin Scale score, 0–2, crude cumulative rates of functional independence, 71.9% tenecteplase vs. 70.5% alteplase, risk difference 2% (95% CI, −3 to 6%)] (31). Although no definitive conclusions about the clinical benefit of tenecteplase can be drawn from our findings, the well-established strong relationship between earlier reperfusion and better functional outcomes (28, 32) suggests that tenecteplase could improve outcomes. Nonetheless, larger studies are needed to detect treatment effect differences between the two thrombolytic agents, given the likely larger effect of endovascular therapy. Despite this, our findings corroborate the accumulating evidence that suggests the superiority of tenecteplase compared to alteplase in large-vessel occlusion strokes. Although the alteplase data were extracted from our prospective Basilar Artery Treatment and Management (BATMAN) registry (38) and the use of early-generation thrombectomy devices and learning curve of interventionalists may have influenced our secondary outcomes (e.g., 90 days modified Rankin scale score), these factors should not influence the primary outcome of reperfusion on the initial angiogram prior to endovascular therapy. Other factors such as time from thrombolysis to reperfusion assessment, which in our study was in favor of the alteplase group, thrombus location, and permeability appear to be independently associated with reperfusion (39). A recently published meta-analysis including only patients with large-vessel occlusions (four studies, *n* = 433 patients) (40) showed that patients receiving tenecteplase had higher successful recanalization (odds ratio, 3.05 [95% CI, 1.73–5.40]), higher odds of good outcomes (modified Rankin Scale scores of 0 to 2, odds ratio, 2.06 [95% CI, 1.15–3.69]), and functional improvement defined as a one-point decrease across all modified Rankin Scale (common odds ratio, 1.84 [95% CI, 1.18–2.87]) at 3 months compared with patients receiving alteplase (40).

Importantly, recent randomized controlled trials failed to show the superiority of endovascular therapy compared to standard medical therapy alone in patients with basilar artery occlusion (41, 42). In the recently completed BASilar artery International Cooperation Study (BASICS) trial, the benefit of endovascular therapy was demonstrated only in patients with moderate-severe clinical syndromes (NIHSS ≥ 10) (43). This suggested that thrombolysis might be the optimal treatment in those with milder deficits.

## Advantages Of Tenecteplase Over Alteplase

The reduced cost and single-bolus administration of tenecteplase vs. an hour-long alteplase infusion in patients with basilar artery occlusion who are being transported between hospitals is a major practical advantage. Tenecteplase is given as a single, 5-s intravenous bolus that requires ~2 min to prepare and administer, whereas alteplase requires preparation of both a bolus syringe containing 10% of the dose and an intravenous pump for infusion of the remaining 90% of the dose over 60 min. Moreover, the use of tenecteplase can minimize the risk of error in the preparation and delivery of the thrombolytic drug in the acute setting. Therefore, tenecteplase could be administered more efficiently in patients with basilar artery occlusion, permitting faster commencement of subsequent endovascular therapy, especially in patients treated with intravenous thrombolysis at primary stroke centers, and then transferred for endovascular therapy (“drip and ship patients”). These patients may have tenecteplase administered at the primary hospital and then be immediately transferred by a standard ambulance without having to wait for critical care transport with staff expert in continuous infusion pump management and without risking interruption or discontinuation of the alteplase infusion during transit (31). Tenecteplase also only requires one drug vial compared to potentially multiple for alteplase (patients >55 kg need two vials of alteplase but only ever one vial of tenecteplase, regardless of patient weight) and is cheaper than alteplase in most countries. Economic analysis of the EXTEND-IA TNK trial indicated that tenecteplase was dominant (cost-saving) vs. alteplase in patients with large-vessel occlusion (44). Finally, tenecteplase has been reported as a more practical thrombolytic agent during the COVID-19 pandemic. Eliminating the alteplase 1-h infusion and the required dedicated second intravenous cannula may reduce staff time in proximity to the patient. Moreover, tenecteplase does not need the intravenous infusion pump that accompanies the patient through other hospital departments and wards, presenting an additional surface that could facilitate the transmission of the virus (45).

Endovascular therapy is highly effective but resource-intensive, and access is currently limited in most countries. Endovascular patients with basilar artery occlusion can be referred to a comprehensive stroke center, either from regional hospitals where there are significant barriers to treatment or from metropolitan hospitals that do not have endovascular therapy capacity. Therefore, there may be significant delays to the initiation of endovascular therapy due to inter-hospital transfer times, especially for patients with basilar artery occlusion who often require intubation before endovascular therapy can be performed. Given that each minute reduction in door-to-reperfusion time is associated with a saving of 4.4 disability-adjusted life days (46), tenecteplase may be a safe and effective treatment to “buy some time” until endovascular therapy can be performed in these patients, especially in those transferred from regional areas. The EXTEND-IA TNK (part II trial) (28) demonstrated that longer times between thrombolysis with tenecteplase and commencement of endovascular therapy in rural sites was associated with significantly higher reperfusion rates prior to endovascular therapy compared with metropolitan patients. Therefore, tenecteplase may allow treatment of a higher number of patients with a devastating form of stroke such as basilar artery occlusion in regional areas and obviate the need to transfer some patients if there is rapid recanalization with early clinical improvement. During inter-hospital transfers, tenecteplase will have time to act on the occlusion which may facilitate early recanalization and have beneficial effects during transfer to a comprehensive center for endovascular therapy.

## Conclusions

Tenecteplase has several practical advantages compared to alteplase. Although randomized controlled trials are needed to detect treatment effect differences between the two thrombolytic agents in patients with basilar artery occlusion, evidence from observational data suggests that it may be associated with higher reperfusion rates prior to thrombectomy, analogous to anterior circulation large-vessel occlusion stroke. Tenecteplase can be considered as an alternative to alteplase in patients with basilar artery occlusion, particularly in “drip and ship” patients.

## Author Contributions

FA has drafted the manuscript and led submission process. BC revised the manuscript for intellectual content. All authors contributed to the article and approved the submitted version.

## Conflict of Interest

The authors declare that the research was conducted in the absence of any commercial or financial relationships that could be construed as a potential conflict of interest.

## Publisher's Note

All claims expressed in this article are solely those of the authors and do not necessarily represent those of their affiliated organizations, or those of the publisher, the editors and the reviewers. Any product that may be evaluated in this article, or claim that may be made by its manufacturer, is not guaranteed or endorsed by the publisher.
